# Operative technique for a combined transcaruncular-transconjunctival approach to double-walled orbital fractures

**DOI:** 10.20517/2347-9264.2022.106

**Published:** 2023-04-04

**Authors:** Luke B. Soliman, Julia L. Lerner, Jung Ho Gong, Marcelo Paiva, Nikhil Sobti, Vinay Rao, Albert S. Woo

**Affiliations:** Division of Plastic and Reconstructive Surgery, Warren Alpert Medical School of Brown University, Providence, RI 02905, USA

**Keywords:** Orbital fracture, trauma, transconjunctival, transcaruncular

## Abstract

Double-walled orbital fractures involving the floor and medial wall are commonly encountered in instances of significant midface trauma. Operative intervention is indicated in the presence of persistent diplopia, significant enophthalmos, or muscle entrapment. Surgical repair of these injuries may be challenging due to large fracture sizes or loss of bony supports. The transconjunctival and transcaruncular approaches have been popularized to reconstruct isolated floor and medial wall fractures, respectively. However, surgical approaches for fractures involving both these walls have not been well described in the literature. In this technical note, we detail a combined transcaruncular-transconjunctival approach that is safe, effective, and aesthetically sensitive.

## INTRODUCTION

Orbital fractures are among the most common facial fractures evaluated within emergency departments nationwide, accounting for approximately 10% to 25% of all maxillofacial trauma^[1]^. Fractures may involve one or more of the orbital walls, resulting in visual disturbances such as diplopia, or adverse cosmetic sequelae such as enophthalmos or hypoglobus^[2,3]^. Fractures of the orbital floor are most common, often requiring surgery in instances of persistent diplopia, acute enophthalmos, muscle ischemia, and significant orbital volume expansion. In as many as 53% of orbital floor fractures, the medial wall may also be involved^[4]^. Compared to isolated floor fractures, injuries involving both the floor and medial wall are more likely to demonstrate diplopia, enophthalmos, or significant volume change, thereby requiring surgical repair^[5]^.

A variety of approaches have been established for access to the floor and medial wall individually. Approaches to the floor include transconjunctival, subciliary, subtarsal, and infraorbital. In contemporary practice, access to the orbital floor is most often achieved by a transconjunctival approach due to low rates of complication and the absence of an external cutaneous incision^[6,7]^. Access to the medial orbital wall may be achieved through trans- (or retro-) caruncular, Lynch, open sky, or coronal approaches. Similar to the transconjunctival approach, transcaruncular incisions are now favored because they minimize the potential for external scarring while supplying direct and reliable access to the medial orbital wall^[8]^.

Surgical repair of double-walled fractures involving both the orbital floor and medial wall may be particularly challenging due to their larger size and loss of internal bony supports. In this circumstance, a unified incision that effectively visualizes fractured areas while maintaining safety and aesthetic considerations can be particularly beneficial. The two aforementioned incision types may be coupled in a combined transcaruncular-transconjunctival approach to supply the benefits of these two methods through a single incision. While numerous reports discuss this access, a technical description of this method is not well expounded in the literature. Herein, we delineate our preferred combined transcaruncular-transconjunctival approach for the repair of orbital injuries involving the medial wall and floor.

## TECHNICAL NOTE: OUR COMBINED TRANSCARUNCULAR-TRANSCONJUNCTIVAL APPROACH

### Transcaruncular access of the medial orbital wall

**Figure 1.**
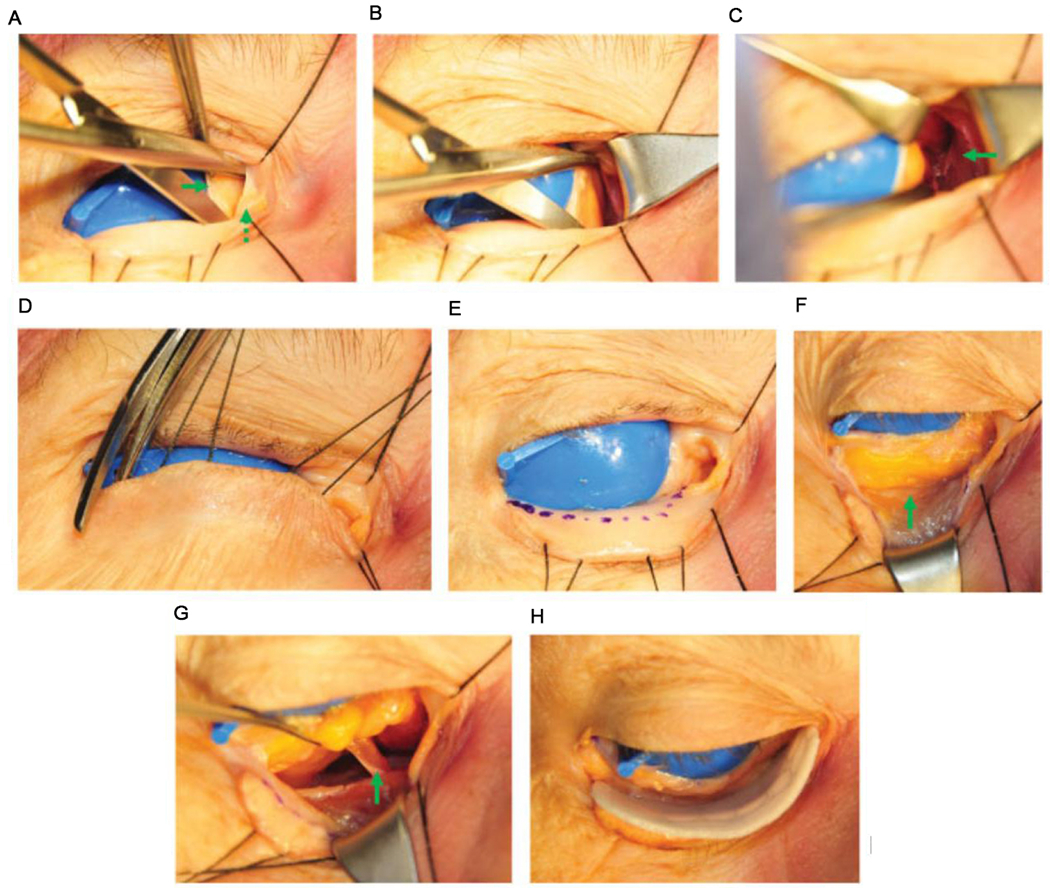
Demonstration of combined transcaruncular-transconjunctival approach in the right orbit of the cadaver. (A) An incision is made between the caruncle (dashed arrow) and semilunar fold (solid arrow) as each structure is retracted. (B) Spreading dissection is performed towards the posterior lacrimal wall. (C) The globe is retracted laterally and subperiosteal dissection behind the lacrimal bone exposes the medial orbital wall (arrow). (D) A medialized lateral canthotomy is performed by incising the inferior canthal crus. (E) The planned transconjunctival incision is marked along the lower lid (dashed line). (F) The inferior orbital rim (arrow) is exposed following transconjunctival incision and retroseptal dissection. (G) The inferior oblique muscle (arrow) is identified as the globe retracts superiorly. (H) A titanium reinforced porous polyethylene implant is positioned through the combined transcaruncular-transconjunctival incision. (Re-published from Nguyen *et al.* with permission from SAGE Publishing^[8]^).

The procedure may begin with either the transcaruncular incision or the transconjunctival approach. For the purposes of this discussion, we will begin with a descriptor of transcaruncular access: A corneal shield lubricated with ophthalmic ointment is placed at the beginning of the case. Transcaruncular access is then initiated [Figure 1A]. The caruncle [Figure 1A, dashed arrow] is identified as a papular structure in the medial canthal region situated medial and anterior to the semilunar fold [Figure 1A, solid arrow]. Two 5-0 silk traction sutures are placed in the upper and lower lid tarsus to assist with exposure. The caruncle is retracted medially with forceps, and an incision is then made between the caruncle and the semilunar fold. The posterior lacrimal crest can then be palpated as a prominent bump in the medial orbital wall. A thin malleable retractor is placed posteriorly to this structure, and a Desmarres retractor can be placed medially to optimize visualization. Stevens tenotomy scissors are slid over the retractor and spreading dissection is performed in a plane above Horner’s muscle toward the posterior lacrimal wall [Figure 1B]. The periosteum is incised behind the posterior lacrimal crest, and subperiosteal dissection then allows visualization of the medial orbital wall [Figure 1C, solid arrow]. The globe can be gently retracted with the malleable [Figure 1C] and further subperiosteal dissection is performed with a Cottle or Freer elevator to visualize the fracture.

### Lateral canthotomy and transconjunctival access of the orbital floor

After accessing the medial wall, attention is then turned towards transconjunctival access to the floor. A lateral canthotomy is made through the lateral aspect of the lower lid, 1 to 2 mm medial to the palpebral fissure, using iris scissors directed in an inferolateral direction [Figure 1D]. While transection of the lateral canthal tendon through the palpebral fissure may establish access to the floor and lateral orbit, this approach is also noted to lead to unpredictable healing and potential round eye deformity. A medialized canthotomy in which only the inferior crus is severed provides excellent exposure to the orbital floor and lateral orbital wall while minimizing possible functional and aesthetic complications. Lateral canthotomy is not always necessary for this procedure; however, this maneuver is frequently useful to optimize the insertion of a large orbital implant. The inferior lid is retracted and the planned transconjunctival incision is marked [Figure 1E, dotted line]. Any prominent vessels of the arterial arcade of the lower eyelid traversing the incision line can be cauterized with pinpoint electrocautery. With a Desmarres retractor placed on the lower lid and a malleable retractor retracting the globe, a transconjunctival incision is made using pinpoint electrocautery [Figure 1E]. Cotton-tipped applicators are then used to dissect bluntly in a retroseptal plane to the orbital rim [Figure 1F, solid arrow]. A preseptal dissection can instead be performed based on surgeon preference in lieu of retroseptal access. The periosteum at the orbital rim is then incised with cautery, providing excellent visualization of the orbital floor.

Once both accesses have been achieved, the incision lines are made to connect between the inferior margin of the transcaruncular approach and the medial edge of the transconjunctival incision. This provides a safe dissection plane posterior to the medial canthus and the lacrimal elements. Dissection at this junction is hampered by the presence of the origin of the inferior oblique muscle, which is discussed below. The presence of this muscle will need to be addressed as it may serve as an obstruction to the reconstruction of the fractured orbit. As noted at the beginning of this section, either transconjunctival or transcaruncular access may be performed first, according to surgeon preference.

### Implant placement and key anatomic elements

**Figure 2.**
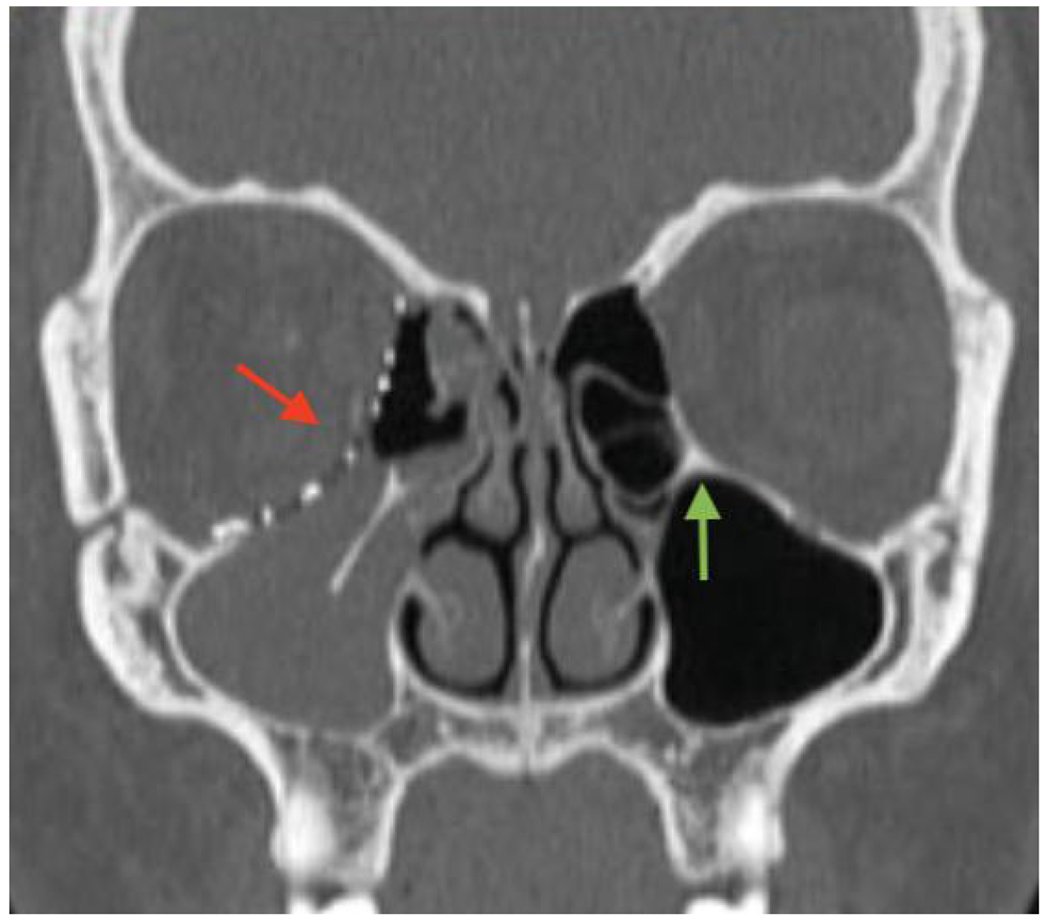
A 31-year-old woman sustained fractures of the right orbital floor and medial wall. Postoperative CT scan demonstrating the absence of internal orbital buttress requiring placement of a single, large implant (red arrow) covering both fractured walls, and intact buttress on contralateral unfractured side (green arrow). (Re-published from Nguyen *et at.* with permission from SAGE Publishing^[8]^).

The orbital floor and medial wall are dissected in a subperiosteal plane using a Cottle periosteal elevator. During dissection of the periorbita and implant placement, special attention must be paid to the inferior oblique muscle, which originates along the inferomedial aspect of the orbital rim and extends obliquely towards the globe [Figure 1G, solid arrow]. Exceptional care must be taken to avoid injury to this structure, which could result in cyclotorsional displacement and functional strabismus. When this structure interferes with implant placement, two separate, smaller implants may instead be attempted for maneuvering and positioning around the muscle, given the presence of a stabilizing internal orbital buttress (IOB). The IOB is a key anatomic landmark of the posteromedial orbit located at the union of the medial wall of the maxillary sinus, the medial wall of the orbit, and the orbital floor [Figure 2, green arrow]^[9,10]^. The articulation of these three boundaries forms a bony septum known as the IOB. When present, the orbital floor and medial wall may be treated as two separate fractures rather than as a single entity. In doing so, two individual prostheses for the floor and medial wall may be stabilized to this segment. This avoids the need to disturb the inferior oblique muscle, allowing the surgeon to operate on either side of the oblique during implant insertion. In instances of severe trauma, however, the IOB may collapse, and placement of a single, larger implant is necessary to span the entire fracture area [Figure 2, red arrow].

**Figure 3.**
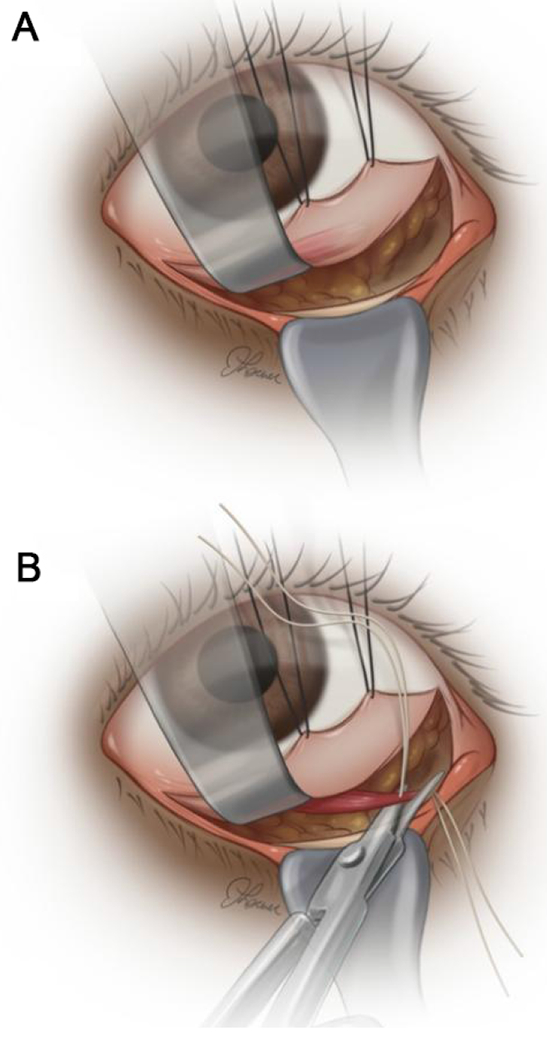
Surgical illustrations of (A) subperiosteal elevation of inferior oblique muscle with periorbita; and (B) division and tagging of muscle with suture.

If an IOB is no longer intact, the obstructing inferior oblique must be mobilized to facilitate implant placement. When possible, this muscle is elevated with the periorbita [Figure 3A]. However, it has been the senior author’s experience that the muscle origin is not tightly adherent to the periosteum in all circumstances. In these instances, the inferior oblique muscle may be tagged for later reapproximation with 5-0 Vicryl (Ethicon, Somerville, NJ) sutures and amputated close to its origin [Figure 3B]. At the time of closure, the tagging sutures of the muscle belly and amputated stump are sutured together to reapproximate the muscle.

Herniated orbital contents are dissected from the maxillary and ethmoid sinuses and returned into the orbit. Wide dissection must be performed to clearly expose the fractures along each of the walls of injury. Establishment of the fracture dimensions should be determined by CT imaging, and these measures are confirmed intraoperatively with a ruler. An appropriately sized implant is chosen for reconstruction. A number of options exist for the reconstruction of complex double-walled orbital fractures. These include traditional flat titanium mash, 3-dimensional anatomic implants, titanium impregnated porous polyethylene implants [e.g., Medpor Titan (Stryker, Kalamazoo, MI); Synpor (DePuy Synthes, West Chester, PA)] or custom orbital reconstruction plates. Once the material is chosen, the implant may be trimmed to the appropriate size and bent or adjusted to simulate the curve between the orbital floor and medial orbit. Autologous implants have been previously described; however, alloplastic materials are favored due to their ready availability and capacity for customization into 3-dimensional shapes. Similarly, while simple porous polyethylene implants remain an option, it is the authors’ opinion that this material is less suited for the reconstruction of complex three-dimensional shapes.

**Figure 4.**
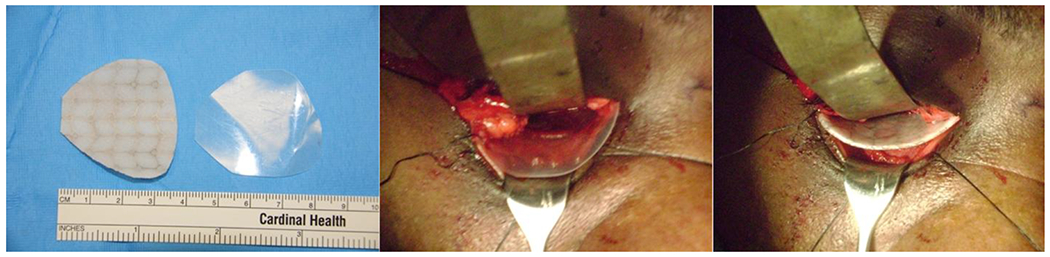
(Left) Titanium impregnated porous polyethylene implant and clear barrier sheet nylon sheet. (Center) Insertion of clear nylon sheet as a barrier to orbital contents prior to prosthesis placement. (Right) Implant placement with nylon sheet removed.

Once the appropriate implant is chosen and prepared, it must be carefully inserted into the large orbital defect, with the margins of the implant resting gently against each of the walls of the fracture. This can be an extremely challenging task, as the orbital contents may be edematous from the recent trauma, creating significant resistance to retraction of these contents back into the globe. The insertion of the implant itself may also be made difficult by the presence of any sharp edges, which may catch onto herniated orbital contents, restricting smooth insertion and making precise placement of the prosthesis difficult. In the senior author’s experience, this occurs most often with titanium-based implants. To facilitate implant placement in these circumstances, a clear nylon sheet [e.g., SupraFOIL (S. Jackson, Inc., Alexandria, VA)] may initially be placed to uphold orbital contents and serve as a barrier as the implant is placed inferiorly to cover the fractured area [Figure 4]. The nylon sheet is subsequently removed following the insertion of the prosthesis

Positioning is visually confirmed so that the implant appropriately rests on stable bony ledges on all sides [Figure 1H]. Some surgeons have advocated intraoperative CT scanning at this time to confirm the anatomic nature of the repair^[11]^. The implant is secured to the orbital rim with a single, self-drilling screw. The corneal shield is removed and a forced duction test is performed. After reassurance that there is no orbital entrapment, the corneal shield is replaced. The operative field is irrigated, and meticulous hemostasis is achieved.

### Closure

**Figure 5.**
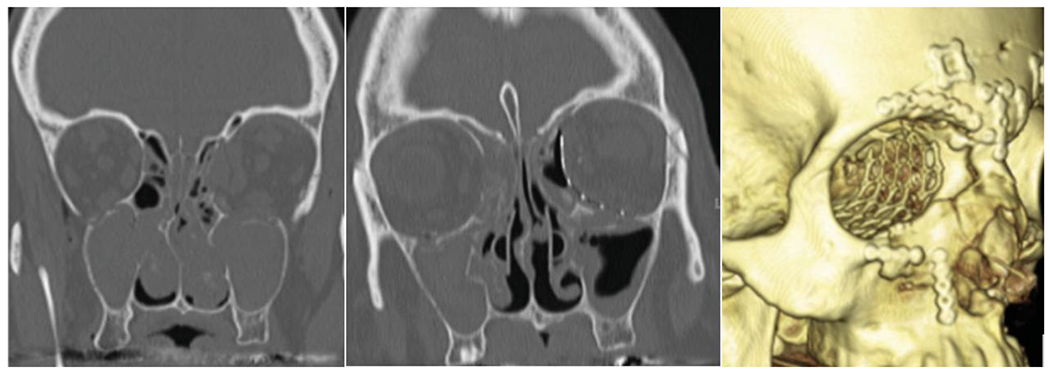
A 65-year-old woman presenting with panfacial fractures underwent a combined transcaruncular-transconjunctival repair of a double-walled orbital fracture. The left-sided floor and medial wall fracture area measured 9.3 cm^2^. Preoperative scan (left), postoperative scan (middle), and postoperative 3D rendering are shown. (Re-published from Nguyen *et al.* with permission from SAGE Publishing^[8]^).

Closure of the transconjunctival, transcaruncular, and lateral canthotomy incisions is performed with buried 6-0 plain gut sutures. The caruncle and conjunctiva are similarly closed with 6-0 plain gut sutures in a buried, interrupted fashion. The lower lid tarsus is reapproximated with a single 5-0 Vicryl suture. This is left loose and is tightened after the conjunctival incision is closed. Upon doing so, the gray line is reapproximated and the cutaneous lower eyelid is repaired, again with plain gut suture. All traction sutures in the caruncle and septum are removed. As necessary, a 5-0 silk suture in the lower lid tarsus may be taped to the forehead and used as a Frost stitch, especially when chemosis is notable. Computed tomography (CT) images of a patient following repair are shown [Figure 5].

## DISCUSSION

Combined fractures of the orbital floor and medial wall are challenging to repair due to difficulty in obtaining adequate exposure to both fractured areas. Individually, the transcaruncular and transconjunctival incisions supply excellent visualization to the medial orbital wall and floor, respectively^[12]^. Therefore, they are commonly used to repair isolated fractures of the orbital floor and lamina papyracea. When combined through a unified technique, these incisions supply a continuous line of access to the inferomedial orbit and broad surgical exposure to the most frequently injured components of the orbit. The combined technique also avoids an external cutaneous incision and possible scarring. Theoretical disadvantages may include scleral show, entropion, disruptions to the nasolacrimal system, and injury to the inferior oblique musculature. However, previous studies note that these rates of complication remain low^[8,13–15]^.

Indications for repair of double-walled fractures are similar to the established indications for isolated fractures of the floor or medial wall. Typically, these include (1) persistent diplopia; (2) entrapment of extraocular muscles; (3) entrapment of periorbital soft tissue; (4) acute enophthalmos (> 2 mm); or (5) large bony defects (> 50% of the floor or 1 cm^3^). When any of these surgical indicators are present in instances of double-walled fractures, a combined transcaruncular-transconjunctival approach should be considered during operative planning^[8,16]^.

Several studies have reported the safety and efficacy of the combined transcaruncular-transconjunctival approach. Nguyen *et al.* concluded that the combined incision for concurrent orbital floor and medial wall fractures provided excellent exposure of all fractured areas without intraoperative complications^[8]^. Postoperatively, 88% of patients underwent CT and all implants were found to remain in good position at a minimum follow-up duration of 3 months. Additionally, there were no complications noted involving the nasolacrimal system, extraocular muscles, or conjunctiva. Shahzad *et al.* assessed the clinical and radiographic data of 7 patients who underwent the combined approach for combined orbital floor and medial wall defects^[13]^. All patients presented with either acute enophthalmos or vertical orbital dystopia preoperatively. All symptoms were adequately resolved after repair using a combined approach. Lee *et al.* conducted a retrospective review of 23 patients who underwent the same method and found that the technique allowed adequate exposure, facilitating placement of a large implant and coverage of the fracture site^[14]^. Despite large defect sizes preoperatively, the authors reported minimal postoperative enophthalmos (mean, 0.17 mm) at a mean follow-up duration of 8.5 months. Similarly, Scolozzi reported that among to patients undergoing the combined approach for severe medial orbital wall fractures, no patient developed postoperative enophthalmos^[15]^.

Beyond the established indications for surgical repair mentioned above, operative interventions for double-walled fractures should also be considered in instances when late visible deformities may be present. Alinasab *et al.* assessed the prospective cosmetic and functional outcomes of patients with double-walled fractures who did not undergo operative management^[16]^. Late visible deformities developed when soft tissue herniation at the time of injury was greater than 0.9 mL. This cutoff may serve as an additional indicator for operative intervention to prevent late deformities.

While the transcaruncular and transconjunctival incisions have been studied individually for isolated wall fractures, they have only been evaluated in combination in a few studies with small sample sizes. Larger studies comparing the combined incision with other established surgical approaches may be indicated to thoroughly evaluate safety and efficacy.

## CONCLUSION

Double-walled orbital fractures commonly require surgical repair, yet access to fractured areas remains a challenge. Previously described approaches may supply access to either the medial wall or floor individually, but access to both walls through a unified approach is not adequately detailed. Herein, we expound techniques for a combined transcaruncular-transconjunctival incision, which safely and effectively exposes the orbital floor and medial wall for surgical repair.

## Data Availability

Not applicable.

## References

[1] Roth FS, Koshy JC, Goldberg JS, Soparkar CN. Pearls of orbital trauma management. Semin Plast Surg 2010;24:398–410. 22550464 10.1055/s-0030-1269769 PMC3324224

[2] Shokri T, Alford M, Hammons M, Ducic Y, Sokoya M. Management of orbital floor fractures. Facial Plast Surg 2019;35:633–9. 31783420 10.1055/s-0039-1700852

[3] Cruz AA, Eichenberger GC. Epidemiology and management of orbital fractures. Curr Opin Ophthalmol 2004;15:416–21. 15625903 10.1097/01.icu.0000136113.56288.87

[4] Thiagarajah C, Kersten RC. Medial wall fracture: an update. Craniomaxillofac Trauma Reconstr 2009;2:135–9. 22110807 10.1055/s-0029-1224775 PMC3052654

[5] Jank S, Schuchter B, Emshoff R, Clinical signs of orbital wall fractures as a function of anatomic location. Oral Surg Oral Med Oral Pathol Oral Radiol Endod 2003;96:149–53. 12931086 10.1016/s1079-2104(03)00317-2

[6] Kesselring AG, Promes P, Strabbing EM, van der Wal KG, Koudstaal MJ. Lower eyelid malposition following orbital fracture surgery: a retrospective analysis based on 198 surgeries. Craniomaxillofac Trauma Reconstr 2016;9:109–12. 27162565 10.1055/s-0035-1567813 PMC4858425

[7] Oztel M, Goh R, Hsu E. Subtarsal versus transconjunctival approach: a long-term follow-up of esthetic outcomes and complications. J Oral Maxillofac Surg 2021;79:1327.e1–6. 10.1016/j.joms.2021.02.004 33684379

[8] Nguyen DC, Shahzad F, Snyder-Warwick A, Patel KB, Woo AS. Transcaruncular approach for treatment of medial wall and large orbital blowout fractures. Craniomaxillofac Trauma Reconstr 2016;9:46–54. 26889348 10.1055/s-0035-1563390 PMC4755730

[9] Kunz C, Audigé L, Cornelius CP, Buitrago-Téllez CH, Rudderman R, Prein J. The comprehensive AOCMF classification system: orbital fractures - level 3 tutorial. Craniomaxillofac Trauma Reconstr 2014;7:S092–102. 25489393 10.1055/s-0034-1389562 PMC4251722

[10] Dreizin D, Nam AJ, Diaconu SC, Bernstein MP, Bodanapally UK, Munera F. Multidetector CT of midfacial fractures: classification systems, principles of reduction, and common complications. Radiographics 2018;38:248–74. 29320322 10.1148/rg.2018170074

[11] Borad V, Lacey MS, Hamlar DD, Dresner HS, Yadava GK, Schubert W. Intraoperative imaging changes management in orbital fracture repair. J Oral Maxillofac Surg 2017;75:1932–40. 28599123 10.1016/j.joms.2017.05.002

[12] Wolkow N, Freitag SK. Transconjunctival and transcaruncular approaches to the orbit. J Neurol Surg B Skull Base 2020;81:422–34. 33072482 10.1055/s-0040-1713849 PMC7561462

[13] Shahzad F, Woo A, Snider-warwick A. The transcaruncular approach for treatment of medial wall and giant orbital blowout fractures. Blast Reconstr Surg 2011;128:31. 10.1055/s-0035-1563390 PMC4755730 26889348

[14] Lee CS, Yoon JS, Lee SY. Combined transconjunctival and transcaruncular approach for repair of large medial orbital wall fractures. Arch Ophthalmol 2009;127:291–6. 19273792 10.1001/archophthalmol.2009.5

[15] Scolozzi P Reconstruction of severe medial orbital wall fractures using titanium mesh plates placed using transcaruncular-transconjunctival approach: a successful combination of 2 techniques. J Oral Maxillofac Surg 2011;69:1415–20. 21272975 10.1016/j.joms.2010.07.015

[16] Alinasab B, Borstedt KJ, Rudström R, New algorithm for the management of orbital blowout fracture based on prospective study. Craniomaxillofac Trauma Reconstr 2018;11:285–95. 30574272 10.1055/s-0038-1641714 PMC6224287

